# Factors Associated With Nonunion After Cervical Fusion Surgery

**DOI:** 10.7759/cureus.50866

**Published:** 2023-12-20

**Authors:** Hiroyuki Inose, Takuya Takahashi, Yu Matsukura, Jun Hashimoto, Kurando Utagawa, Satoru Egawa, Kentaro Yamada, Takashi Hirai, Toshitaka Yoshii

**Affiliations:** 1 Department of Orthopedics, Dokkyo Medical University Saitama Medical Center, Saitama, JPN; 2 Department of Orthopedics, Tokyo Medical and Dental University, Tokyo, JPN

**Keywords:** glycated hemoglobin (hba1c), intact parathyroid hormone, anterior cervical decompression and fusion, posterior cervical decompression and fusion, nonunion

## Abstract

Background

Bony fusion is a critical factor in the outcome of cervical spinal fusion surgery. While several factors have been proposed to affect bony fusion, their relative importance remains unclear. This study aimed to investigate the factors associated with bony fusion after cervical spinal fusion surgery.

Methods

We retrospectively evaluated the significance of the various factors on bone fusion after cervical fusion surgery. Then, multivariate logistic regression analyses were performed to determine the independent factors associated with bony fusion. A receiver operating characteristic (ROC) analysis was performed to evaluate the cutoff threshold.

Results

This study included a total of 52 patients with a mean age of 64 years. Among them, 28 (54%) were male, and 39 (75.0%) achieved bony fusion. The multivariate logistic regression analysis showed that preoperative intact parathyroid hormone (PTH) levels (odds ratio, 1.04; 95% confidence interval, 1.01-1.08; p-value = 0.02) and hemoglobin A1c (HbA1c) levels (odds ratio, 2.87; 95% confidence interval, 1.07-8.74; p-value = 0.04) were significant factors associated with bony fusion after cervical fusion surgery. The optimum cutoff values of intact PTH and HbA1c were 63.5 pg/mL and 6.2%, respectively.

Conclusions

This study identified preoperative intact PTH and HbA1c as significant factors associated with bony fusion after cervical fusion surgery. These biomarkers can be used to identify patients at higher risk of nonunion to optimize patient conditions and guide preoperative and postoperative management strategies.

## Introduction

Cervical fusion surgery is a common procedure for patients with degenerative cervical spinal diseases or spinal instability [1,2]. Successful bone fusion is crucial for achieving satisfactory clinical outcomes and preventing complications such as hardware failure and nonunion [3,4]. However, the factors that influence bone fusion after cervical fusion surgery are not well understood.

Several factors have been identified that may affect bone fusion after spinal fusion surgery. Age, smoking status, poor nutrition, bone metabolism, and comorbidities have been suggested as potential predictors of bone fusion outcomes [3,5-9]. However, the results of these studies are inconsistent and inconclusive. Differences in results between these studies may also arise from factors such as variations in the surgical target's location, whether it is the cervical or lumbar spine, as well as enhancements in fusion rates attributed to recent advances in instrumentation. For instance, some studies have found that smoking status is associated with decreased bone fusion rates [5,7,8], while others have not [6,9]. Despite the extensive research on the factors associated with bone fusion after cervical spinal fusion surgery, several important questions remain unanswered. Specifically, the specific factors that contribute to successful bone fusion after cervical fusion surgery are not well established. Moreover, the relative importance of different factors and their interactions are unclear.

To address these knowledge gaps, we conducted a retrospective study to investigate the factors associated with bone fusion after cervical fusion surgery. We examined the relationship between patient characteristics, surgical factors, and bone fusion outcomes using multivariate regression analyses.

## Materials and methods

Study population

From August 2019 to August 2021, surgery was performed on 132 consecutive patients diagnosed with degenerative cervical myelopathy (DCM) at Tokyo Medical and Dental University. The exclusion criteria included a history of prior cervical surgery, renal failure (with an estimated glomerular filtration rate {GFR} of <35 mL/minute/1.73 m²), and incomplete laboratory or imaging data. In total, 41 patients were excluded from the study. Furthermore, patients who had undergone cervical artificial disc replacement (two patients) or laminoplasty (39 patients) were excluded from the perspective of this study's assessment of bone fusion. Demographic information, including age, gender, the number of operated segments, and the etiology of myelopathy, was gathered. The determination of surgical indications and procedures (including anterior decompression and fusion, posterior decompression and fusion, and combined anterior and posterior decompression and fusion) was based on individual patient factors. These factors included the patient's neurological condition, the presence of anterior compression, and spinal alignment.

Serum measurements

Prior to the surgical procedure, blood samples were obtained from patients in the outpatient clinic. The assessment of bone remodeling in these patients involved the measurement of specific markers of bone turnover, namely, procollagen type 1 amino-terminal propeptide (P1NP) for evaluating bone formation and tartrate-resistant acid phosphatase 5b (TRACP-5b) for assessing bone resorption. These markers were selected due to their low diurnal variability and their independence from the influences of fasting or renal dysfunction [10,11]. In order to characterize the individual bone turnover balance of the patients, we computed a parameter known as the bone turnover ratio (BTR) [6]. The BTR was determined by dividing the serum concentration of TRACP-5b by the serum concentration of P1NP, as previously described [6]. In addition to the aforementioned assessments, we also evaluated serum albumin as an indicator of nutritional status, hemoglobin A1c (HbA1c) as a marker of glucose tolerance, and calcium levels, estimated GFR, serum 25-hydroxyvitamin D (25(OH)D), and intact parathyroid hormone (PTH).

Additionally, we explored the association between fusion status and various clinical factors, such as age, gender, smoking history, and the number of operated segments.

Outcome measures

The assessment of outcomes was conducted both prior to and one year following the surgical procedure, utilizing the Japanese Orthopedic Association score for the assessment of cervical myelopathy (C-JOA score) as a means to evaluate cervical myelopathy [12]. The C-JOA score encompasses the evaluation of six functional categories, which include motor dysfunction in the upper and lower extremities, sensory function in the upper and lower extremities and trunk, and bladder function. Each of these subscales is assigned a score ranging from 0 to 4, 4, 2, 2, 2, and 3, respectively [12]. The minimum score possible is 0, while the overall maximum score achievable is 17 [13]. The recovery rate of the C-JOA score was calculated using Hirabayashi's method, applying the following formula: recovery rate (%) = (postoperative C-JOA score - preoperative C-JOA score) × 100 / (17 - preoperative C-JOA score) [14]. A computed tomography (CT) performed one year postoperatively was used to assess bony union. Failed union was defined as the existence of lucency at the fusion device or pedicle screw margins [15]. For the 15 patients for whom postoperative CT was not taken, fusion status was determined by flexion and extension X-ray according to the published radiographic fusion criteria (interspinous motion of <1 mm) [16].

Statistical analysis

After assessing data normality through the Shapiro-Wilk test, we proceeded to perform a Wilcoxon signed-rank test to investigate potential differences in the C-JOA score between the preoperative and one-year postoperative periods. To compare the union and nonunion groups in terms of gender, etiology, surgical procedure, and history of cigarette smoking, we utilized Fisher's exact test. Mann-Whitney U test was used to analyze other data. In order to identify the most significant risk factors associated with nonunion, a risk factor analysis was conducted using multivariate logistic regression analysis, employing a forward-backward stepwise procedure. First, predictors that exhibited a p-value of ≤0.25 in the univariate analysis were selected for inclusion in the model [17]. Second, a stepwise model selection procedure was implemented among these potential candidates. Predictors with a p-value of >0.1 were removed. To assess the cutoff point, a receiver operating characteristic (ROC) analysis was conducted. Statistical analysis was conducted using JMP version 14 (SAS Institute Inc., Cary, NC), and significance was defined as p-values of <0.05. All data are presented as means + standard deviation (SD).

## Results

Patient demographics

A total of 52 patients were included in this study. The mean age was 64.0 years. The mean preoperative C-JOA score was 10.6 points, demonstrating a significant improvement to an average of 14.3 points at the one-year postoperative mark (p-value < 0.0001). The average recovery rate was calculated at 56.8% (Table 1).

**Table 1:** Baseline characteristics of patients and neurological outcomes. Data are presented as mean ± standard deviation or n (%). HbA1c, hemoglobin A1c; eGFR, estimated glomerular filtration rate; PTH, parathyroid hormone; P1NP, procollagen type 1 amino-terminal propeptide; TRACP-5b, tartrate-resistant acid phosphatase 5b; 25(OH)D, 25-hydroxyvitamin D; CSM, cervical spondylotic myelopathy; OPLL, ossification of posterior longitudinal ligament; ADF, anterior decompression and fusion; PDF, posterior decompression and fusion; C-JOA, Japanese Orthopedic Association score for the assessment of cervical myelopathy

Characteristics	N = 52
Age, years (range)	64.0 ± 10.3 (40-84)
Sex	Male, 28 (54%)
Albumin, g/dL	4.2 ± 0.4
Calcium, mg/dL	9.5 ± 0.3
HbA1c, %	6.1 ± 0.7
eGFR, mL/minute/1.73 m^2^	71.7 ± 21.7
Intact PTH, pg/mL	49.3 ± 20.9
P1NP, ng/mL	45.8 ± 14.8
TRACP-5b, mU/dL	393.3 ± 180.0
25(OH)D, ng/mL	15.0 ± 5.1
Smoking, n (%)	7 (13.5)
Etiology	CSM 19 OPLL 33
Surgical procedure	ADF 29 PDF 21 anterior + posterior 2
Preoperative C-JOA score	10.6 ± 2.7
Postoperative one-year C-JOA score	14.3 ± 2.1
Recovery rate, %	56.8 ± 30.5

Factors associated with nonunion after cervical fusion surgery

We conducted univariate analysis to identify factors that associated with the occurrence of nonunion. Of the 52 patients, 13 (25.0%) were diagnosed with nonunion. The values of intact PTH and HbA1c exhibited a trend of being lower in the bone fusion group compared to the nonunion group (p-value = 0.09 and 0.09, respectively). However, no statistically significant difference was found between the two groups for other factors (Table 2).

**Table 2:** Univariate analysis: difference between union and nonunion groups. HbA1c, hemoglobin A1c; eGFR, estimated glomerular filtration rate; PTH, parathyroid hormone; P1NP, procollagen type 1 amino-terminal propeptide; TRACP-5b, tartrate-resistant acid phosphatase 5b; BTR, bone turnover ratio; 25(OH)D, 25-hydroxyvitamin D; CSM, cervical spondylotic myelopathy; OPLL, ossification of posterior longitudinal ligament; ADF, anterior decompression and fusion; PDF, posterior decompression and fusion; C-JOA, Japanese Orthopedic Association score for the assessment of cervical myelopathy

Characteristic	Union, n = 39	Nonunion, n = 13	P-value
Age, years	63.7 ± 11.0	65.2 ± 8.2	0.76
Sex, female	20	4	0.34
Albumin, g/dL	4.2 ± 0.4	4.2 ± 0.3	0.68
Calcium, mg/dL	9.5 ± 0.3	9.4 ± 0.3	0.32
HbA1c, %	6.0 ± 0.6	6.4 ± 0.8	0.09
eGFR, mL/minute/1.73 m^2^	72.6 ± 24.0	69.0 ± 12.6	0.82
Intact PTH, pg/mL	45.6 ± 17.3	60.3 ± 27.0	0.09
P1NP, ng/mL	46.4 ± 15.4	44.0 ± 13.1	0.66
TRACP-5b, mU/dL	379.9 ± 173.2	433.5 ± 200.9	0.49
25(OH)D, ng/mL	15.1 ± 5.1	14.8 ± 5.3	0.95
BTR	8.4 ± 2.9	10.2 ± 4.4	0.20
Operated segments	3.5 ± 1.7	4.0 ± 1.6	0.44
Smoking status	7	0	0.17
Etiology	CSM 14 OPLL 25	CSM 5 OPLL 8	＞0.99
Surgical procedure	ADF 22 PDF 16 anterior + posterior 1	ADF 7 PDF 5 anterior + posterior 1	0.73
Preoperative C-JOA score	10.6 ± 2.8	10.8 ± 2.2	0.79
Postoperative one-year C-JOA score	14.3 ± 2.2	14.4 ± 1.8	0.95
Recovery rate, %	56.1 ± 32.5	59.0 ± 24.7	0.84

We then conducted a stepwise logistic regression analysis to identify independent factors associated with nonunion. In the variable selection process for stepwise logistic regression, factors that exhibited p-values of less than 0.25 in the univariate regression analysis (intact PTH, HbA1c, BTR, and smoking status) were considered as candidate variables. Consequently, intact PTH and HbA1c levels were identified as the independent factors that are associated with nonunion (Table 3).

**Table 3:** Risk factors of nonunion: results of stepwise multivariate logistic regression analysis. *P-value < 0.05. CI, confidence interval; PTH, parathyroid hormone; HbA1c, hemoglobin A1c

Characteristic	Odds ratio	95% CI	P-value
Intact PTH, pg/mL	1.04	1.01-1.08	0.02*
HbA1c, %	2.87	1.07-8.74	0.04*

**Figure 1:**
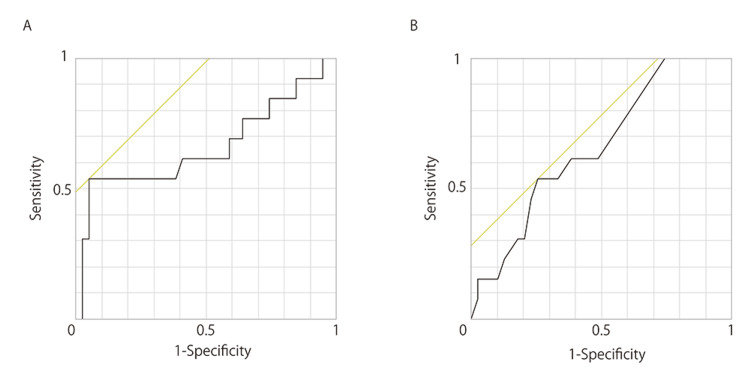
Receiver operating characteristic analysis of intact PTH (A) and hemoglobin A1c (B). The area under the curve, sensitivity, and specificity of intact PTH were 0.66, 0.539, and 0.949, respectively (A). The area under the curve, sensitivity, and specificity of hemoglobin A1c were 0.66, 0.539, and 0.744, respectively (B). PTH: parathyroid hormone

Finally, a receiver operating characteristic (ROC) analysis was performed to evaluate the predictive performance of nonunion. The cutoff level for intact PTH according to the Youden index was 63.5 pg/mL. The area under the curve (AUC) was 0.66. The sensitivity and specificity were 0.539 and 0.949, respectively (Figure 1A). The cutoff HbA1c level according to the Youden index was 6.2%. The AUC was 0.66. The sensitivity and specificity were 0.539 and 0.744, respectively (Figure 1B).

## Discussion

Our retrospective study evaluated the factors associated with nonunion after cervical fusion surgery in 52 patients. The mean preoperative C-JOA score was 10.6 points, and it showed a significant improvement to an average of 14.3 points at one year postoperatively (p-value < 0.0001). The average recovery rate was 56.8%. Of the 52 patients, 13 (25.0%) were diagnosed with nonunion. Stepwise logistic regression analysis identified intact PTH and HbA1c levels as independent factors associated with nonunion. The cutoff values for intact PTH and HbA1c levels in predicting nonunion were 63.5 pg/mL and 6.2%, respectively.

The relevant interpretation of the results of the study is the identification of intact PTH and HbA1c levels as factors associated with nonunion after cervical fusion surgery. Accordingly, patients with higher intact PTH and HbA1c levels may be at increased risk of nonunion and may benefit from the preoperative optimization of these parameters. Previous studies have identified other risk factors, such as older age [18], smoking [5,7], and low vitamin D levels [19], but our study did not find any association between these factors and fusion status. These discrepancies may be due to differences in patient populations, sample size, or study design. To the best of our knowledge, this study is the first to show that higher intact PTH levels were associated with less favorable bone fusion after spinal fusion surgery. Although teriparatide administration promoted bone fusion after posterolateral lumbar fusion in a randomized controlled trial [20], the mechanism by which bone fusion is promoted has remained unclear. As endogenous intact PTH is persistently suppressed by teriparatide administration [21], lowering the intact PTH level may be advantageous for bone fusion after spinal fusion surgery. Because vitamin D3 administration also lowers serum PTH levels [22], the preoperative administration of vitamin D3 to patients with high intact PTH levels (>63.5 pg/mL) may lead to increased bone fusion rates after cervical fusion surgery. Since vitamin D receptors are also present in muscles, the perioperative administration of vitamin D may be useful for restoring muscle strength [23]. We would like to investigate this therapeutic strategy prospectively in the future.

A prospective study found that urinary N-telopeptide (NTX) was higher in the fusion group than in the nonunion group after anterior cervical decompression and fusion [24]. Unfortunately, we did not measure NTX because of its high diurnal variability [25] but measured TRACP-5b as a bone resorption marker. In our study, TRACP-5b was not significantly associated with nonunion (p-value = 0.49), but BTR tended to have an association with nonunion (p-value = 0.20). In addition, the mean BTR was higher in the nonunion group (8.4 versus 10.2), indicating that bone resorption predominates over bone formation, a condition that may be unfavorable for fusion [6]. However, further prospective studies are needed to determine the usefulness of bone metabolism markers in predicting bone fusion after cervical fusion surgery.

Stepwise logistic regression analysis identified HbA1c levels as an independent predictor of nonunion. Regarding the association between hyperglycemia and bone fusion in spinal surgery, retrospective studies have shown that the nonunion rates were significantly higher in diabetic patients than in nondiabetic ones [26,27]. Earlier studies have shown that the dysfunction of both osteoclasts and osteoblasts in diabetes may play a role in the elevated nonunion rate following cervical fusion surgery [28,29]. A retrospective case-control study found that an elevated preoperative or postoperative serum glucose level was independently associated with an increased risk of surgical site infection [30]. Accordingly, the preoperative normalization of blood glucose levels may be advantageous not only in preventing infection but also in preventing nonunion after spinal fusion surgery.

Our study has some limitations. First, this retrospective study relied on medical records for data collection, and forty-one patients were excluded due to criteria such as inadequate blood draws or imaging studies. The limitations related to data collection and patient exclusions could impact the reliability, generalizability, and validity of the findings. Second, there are 15 patients whose bone union was evaluated by anteroposterior flexion X-rays instead of CT. However, a retrospective comparative study showed that the interspinous motion criteria demonstrated a similar accuracy to that of conventional bridging bone criteria on CT scans [16]. Nevertheless, the use of two techniques to assess bone union introduces potential limitations that may affect the accuracy and generalizability of the study results. Third, it should be noted that the AUC for intact PTH and HbA1c did not demonstrate high accuracy. This can likely be attributed to the complex and multifactorial nature of bone fusion following cervical fusion surgery, involving factors such as age, nutritional status, the effectiveness of each instrumentation procedure, and the state of bone metabolism. Therefore, it is crucial to validate these findings through large-scale prospective studies and further evaluate the effectiveness of interventions aimed at optimizing these biomarkers and their impact on union status.

## Conclusions

Our retrospective study identified intact PTH and HbA1c levels as independent predictors of nonunion after cervical fusion surgery, while age and nutritional status showed no association with fusion. These biomarkers can be used to identify patients at higher risk of nonunion and guide preoperative and postoperative management strategies. These findings have important implications for clinicians in optimizing patient conditions to increase the rate of bone fusion in cervical spinal fusion surgery. Further research is warranted to determine the utility of optimizing preoperative patient health, based on these findings, for enhancing union rates following cervical fusion surgery.
